# The livelihood impacts of COVID-19 in urban South Africa: a view from below

**DOI:** 10.1007/s11205-022-02978-7

**Published:** 2022-09-30

**Authors:** Simone Schotte, Rocco Zizzamia

**Affiliations:** 1 United Nations University World Institute for Development Economics Research (UNU-WIDER), Katajanokanlaituri 6 B, FI-00160 Helsinki, Finland; 2 Southern Africa Labour and Development Research Unit (SALDRU), University of Cape Town, Cape Town, South Africa; 3 Mansfield Rd, University of Oxford, OX1 3TB Oxford, UK

**Keywords:** COVID-19, Welfare dynamics, Lockdown, South Africa, Mixed methods, **codes**: I18, J46, O55

## Abstract

This paper investigates the impact of the COVID-19 pandemic and related policy measures on livelihoods in urban South Africa. Using qualitative research methods, we analyse two rounds of semi-structured phone interviews, conducted between June and September 2020 in the township of Khayelitsha, Cape Town. We contextualise these by presenting a snapshot of the nationwide dynamics using quantitative panel data. Our findings describe how the shock of the COVID-19 pandemic has deepened the economic vulnerability which preceded the crisis. Survivalist livelihood strategies were undermined by the economic disruption to the informal sector, while the co-variate nature of the shock rendered social networks and informal insurance mechanisms ineffective, causing households to liquidate savings, default on insurance payments, and deepen their reliance on government grants. In addition, the impact of the pandemic on schooling may deepen existing inequalities and constrain future upward mobility.

## Introduction

The COVID-19 pandemic has delivered a devastating economic shock to livelihoods across the world. Early indications suggest that within countries, the impact of the pandemic has been unequal across households with differential access to income, assets, employment, health care, and social protection, as well as along gender lines (Adams-Prassl et al., 2020; Gisselquist & Kundu, 2020). The inequality of the impact was acutely felt in the labour market, where workers in elementary occupations, those in the urban informal economy, and those without unemployment insurance have been most affected by distancing policies and the overall drop in consumer demand (Balde et al., 2020; Bassier et al., 2021; Espi et al., 2020; Jain et al., 2020a; Lakuma & Nathan, 2020; Ranchhod & Daniels, 2020a; Schotte et al., 2021).

Much of the evidence for these effects has relied primarily on quantitative data collected through rapid telephone surveys. However, to obtain a more granular understanding of the livelihood responses to the COVID-19 shock, there is much to gain by combining quantitative data with an analysis of detailed qualitative evidence. This is the goal of this paper.

We assess how the COVID-19 pandemic and related policy measures have affected people’s livelihoods, focusing on low-income and disadvantaged communities in urban South Africa, with the aim of providing a detailed ‘view from below’. We present a snapshot of the quantitative evidence on the COVID-19 impact that has been gathered at the national level and enrich these findings by providing an in-depth qualitative analysis that explores the perceptions, coping strategies, and main challenges experienced by people who were highly vulnerable to the shock.

We focus on South Africa as a case study. The COVID-19 lockdown in South Africa was one of the earliest and strictest in global comparison (Gustafsson, 2020), causing a substantial disruption of labour markets, with already disadvantaged workers bearing the heaviest burden (Casale & Shepherd, 2020; Espi et al., 2020; Jain et al., 2020a; Ranchhod & Daniels, 2020a; Rogan & Skinner, 2020). Despite stringent confinement policies implemented to reduce contagion, COVID-19 infections in South Africa surged rapidly. Cape Town—with its poor, densely populated townships—and the surrounding Western Cape province quickly emerged as hotspots.

Our qualitative research strategy draws on two rounds of semi-structured interviews conducted between June and September 2020 with respondents residing in Khayelitsha, a large township on the outskirts Cape Town. The sample was drawn from a previous qualitative study—consisting of in-depth life-history interviews and wealth ranking exercises—that we conducted in 2017. The interviews in this extension study focused on the impact of the pandemic on economic livelihoods and well-being. This analysis was supplemented by two key informant interviews that shed light on issues experienced at the broader community level.

Our findings highlight three interrelated consequences of the COVID-19 pandemic. First, consistent with prior quantitative evidence on the COVID-19 shock in South Africa (Jain et al., 2020a; Ranchhod & Daniels, 2020a), we find that the pandemic was experienced first and foremost as a sudden and dramatic shock to labour markets. While this shock to earnings and employment was experienced by almost all workers in our sample, the consequences appear especially severe and long-lasting for those in informal work, whether in wage labour or self-employment. The shock also percolated through to those not directly affected by job or earnings losses, drying up distributional channels of support. Consistent with Jain et al., (2020b), this shock to labour market income appears to have affected household spending, with several respondents reducing consumption of essential food and non-food items.

Second, the shock to earnings has led to a general decrease in the underlying resilience of households to future potential shocks—which could include the second wave of COVID-19 infections from which South Africa emerged in February 2021. Providing novel evidence on a dimension not well captured in the quantitative data, our qualitative data show that households have lost access to both formal and informal mechanisms of social insurance in the crisis. Several respondents reported defaulting on funeral policies, drawing down on savings, witnessing rotating savings and credit associations disintegrate, and losing access to remittance income. Covariate shocks such as the COVID-19 pandemic compromise community-based risk-sharing institutions (Dercon, 2002), and subsequently expose individuals to future idiosyncratic shocks. In this regard, the expansion of government social protection through top-ups to existing grants and through the introduction of a new social relief grant has been indispensable in sustaining the livelihoods of the poor.

Third, amongst our interlocutors, there was a general sense that developments in the pandemic context have led to a perception of a loss of control of the outcomes in one’s life. We propose that the psychological distress experienced by individuals in our sample can be understood in terms of this fatalistic shift. Individual anxieties were centred on where respondents have ‘skin in the game’—younger men were distressed primarily about their perceived loss of agency in the labour market, while older respondents were more anxious about the uncontrollable disease environment.

This work adds to two strands of research. First, we expand on the rapidly expanding body of research investigating the livelihood impacts of COVID-19 in developing countries in general (Abraham et al., 2020; Balde et al., 2020; Gisselquist & Kundu, 2020; Lakuma & Nathan, 2020; Sumner et al., 2020), and in South Africa in particular (Espi et al., 2020; Jain et al., 2020a; Köhler & Bhorat, 2020; Ranchhod & Daniels, 2020a; van der Berg et al., 2020; Wills et al., 2020). By presenting novel qualitative evidence, our paper is able to speak to processes which remain out of reach of large quantitative rapid-assessment surveys—such as the inter-linkages between livelihood strategies and informal support networks, the psychological experience of the pandemic, and the exacerbation of underlying vulnerabilities.

Second, our paper adds to existing work investigating the determinants of economic vulnerability and resilience to shocks, expanding both the qualitative (Neves & Toit, 2013; Du Toit & Neves, 2007) and quantitative (Schotte et al., 2018, 2022) literature. In this regard, the COVID-19 context provides us with the opportunity to investigate how prior work on vulnerability to economic shocks maps onto the outcomes observed in face of new and dramatic health, economic, and social challenges. Previous research has shown that—prior to the pandemic—two-thirds of the South African population were either poor or vulnerable to falling into poverty (Schotte et al., 2018). As Schotte (2019) and Zizzamia (2020) argue, among those households with few buffers to protect their living standards, negative shocks to income can easily generate a poverty trap that is difficult to escape from, and health shocks and job losses are among the main trigger events that can precipitate a downward spiral. Making use of newly collected data in South Africa, we are able to show that pre-existing markers of vulnerability map onto poverty and deprivation outcomes in the post-COVID context, and help explain heterogeneity in the experience of the shock.

Our findings give rise to concerns that the COVID-19 pandemic has both exposed and exacerbated existing inequalities. It may not only present a temporary income shock but also hamper people’s income generating activities in the longer term—with potentially lasting implications for the incidence, depth, and severity of poverty.

The paper proceeds as follows: Sect. 2 discusses the South African context and policy landscape in the wake of the COVID-19 pandemic. Section 3 presents the qualitative and quantitative data used and the methodology of analysis. Section 4 provides a snapshot assessment of the quantifiable economic impact of COVID-19 on South African households. Section 5 proceeds with an in-depth analysis of our qualitative data, assessing the impact of COVID-19 on township livelihoods. Section 6 concludes.

## COVID-19 in South Africa: background and policy environment

For policy-makers around the world, navigating the response to the COVID-19 pandemic has been a balancing act between protecting public health and the economy. In the first months of the pandemic, South Africa bore one of the largest COVID-19 caseloads worldwide, and its policy response was one of the earliest and strictest in global comparison.

After the first case was registered in early March 2020 and in the face of rising infections, a national lockdown came into effect on 27 March. This full lockdown was later framed by the government as ‘Level 5’ in a ‘Risk Adjusted Strategy’ to manage the spread of COVID19. Over time, the government gradually relaxed the regulations, with a move onto ‘Level 4’ coming into effect on 1 May, ‘Level 3’ on 1 June, ‘Level 2’ on 18 August, and ‘Level 1’ on 21 September 2020 (see COVID-19 South African Online Portal (2020) for a summary description of alert levels).

**Fig. 1:**
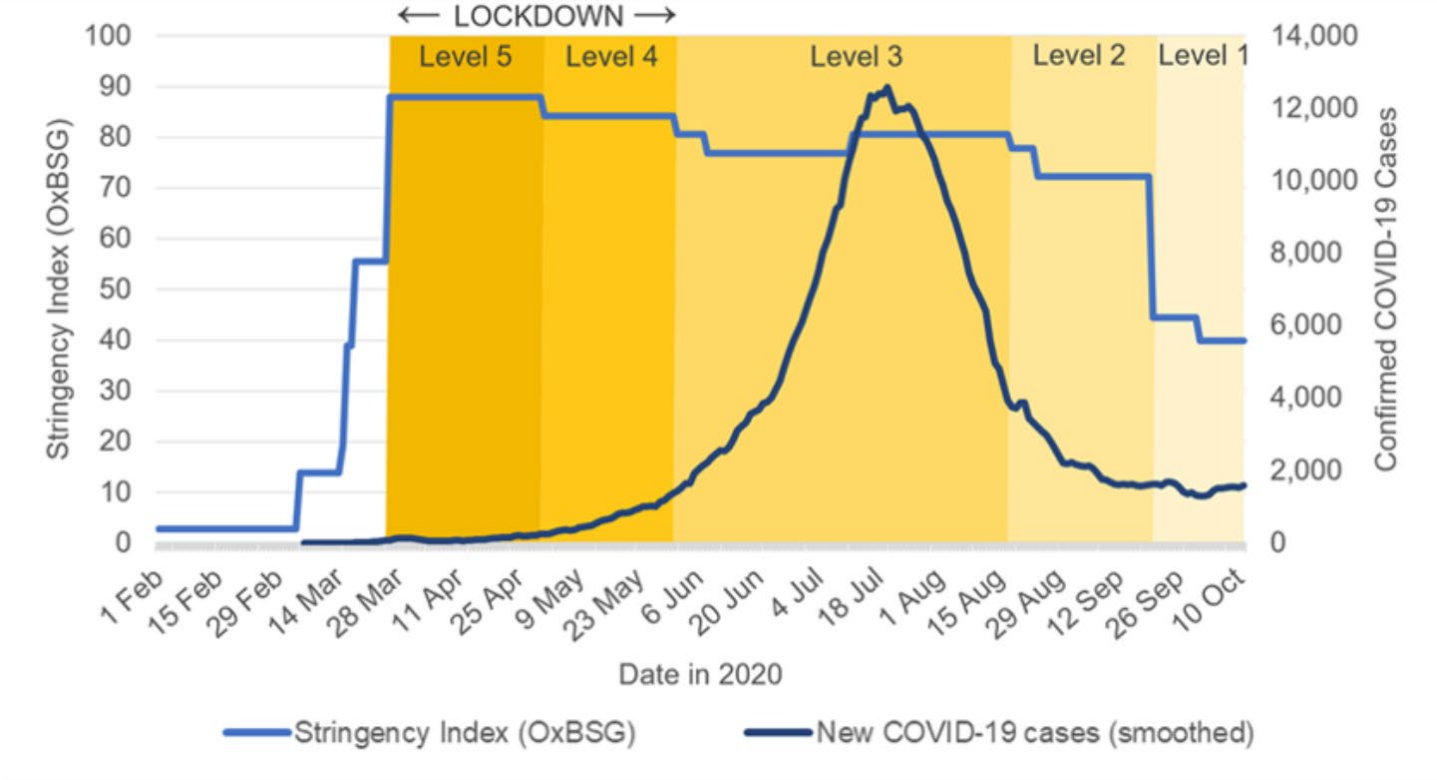
COVID-19 cases and government response stringency index. (Note: the stringency index published by the Blavatnik School of Government (OxBSG) is a composite measure based on nine response indicators including school closures, workplace closures, and travel bans, rescaled to a value from 0 to 100 (strictest); it shows the pandemic response level in the districts subject to the strictest lockdown measures. Source: authors’ illustration based on Hale & Webster (2020) and Roser et al., (2020))

Figure 1 illustrates the stringency of policy measures that were in place in South Africa between March and October 2020 in response to COVID-19. Level 5 entailed a complete stop to all but essential commercial activity and severe curtailment of freedom of personal movement, including strict stay-at-home orders and the active involvement of the South African Defence Force in enforcing regulations. In subsequent levels, restrictions on commercial activity were gradually relaxed, but remained relatively rigid by international standards. Strict stay-at-home orders remained in force in Level 4, so that meaningful relaxation on the freedom of movement for the general population only began in Level 3.

Despite stringent, early confinement policies to reduce contagion, COVID-19 infections in South Africa surged rapidly, with the first wave reaching peak levels in mid-July 2020 (see Fig. 1). In the early phases of the pandemic, Cape Town—with its poor, densely populated townships—and the surrounding Western Cape province quickly emerged as hotspots, accounting for 45 per cent of the nation’s confirmed cases as of 28 June 2020 (NICD 2020). Despite the sharp subsequent fall in new infections between late July and the end of August, by September 2020 South Africa had by far the highest number of total confirmed COVID-19 cases in Africa and the sixth-highest case count worldwide.

The economic impact of stringent distancing policies and the overall drop in demand were acutely felt in the labour market—triggering job losses, business closures, and underemployment. Employment losses were concentrated among those who were already disadvantaged prior to the pandemic—women, less-skilled workers, informal workers, low-income earners, and those with a history of unemployment (Casale & Shepherd, 2020; Espi et al., 2020; Jain et al., 2020a; Ranchhod & Daniels, 2020a; Rogan & Skinner, 2020). The evidence also points to a large impact of the COVID-19 crisis on household poverty and food insecurity (van der Berg et al., 2020).

With the gradual relaxation of confinement measures to Level 4 (1 May) and Level 3 (1 June), commercial activity recommenced and labour markets experienced a partial recovery (Jain et al., 2020b; Ranchhod & Daniels, 2020b). Approximately half of the loss in active employment that occurred between February and April was recovered by June (Jain et al., 2020b), and the recovery was sustained into October (Bassier et al., 2021).

Targeted social assistance measures introduced from May onward also helped to cushion the blow delivered by COVID-19. In response to the crisis, on 26 March 2020, South Africa’s government introduced the Temporary Employee/Employer Relief Scheme (TERS), a social insurance scheme administered through the contribution-based Unemployment Insurance Fund (UIF). Approximately one month later, on 21 April 2020, a set of social assistance measures were announced, aimed at delivering relief to households not covered by employment-related insurance schemes. These consisted of: (a) an increase to the Child Support Grant (CSG) of ZAR300 (US$17)^1^ for one month, followed by an increase of ZAR500 (US$30) per month from June to October (but limited during the latter period to one increase per caregiver); (b) an increase to all other social grants (such as the old age pension and the disability grant) of ZAR250 (US$15) per month until October, and; (c) the introduction of a special COVID-19 Social Relief of Distress Grant (SRD) of ZAR350 (US$21) per month, newly introduced to assist people who are unemployed and not receiving any other grant or UIF (Bassier et al., 2021).

The delivery of UIF-TERS and the SRDG were compromised by delays and early implementation failures. Despite these initial delays, Jain et al., (2020b) show that coverage by the SRDG increased remarkably between June and July/August. By October, the SRDG had become a core element of South Africa’s social assistance landscape and, alongside the CSG, proved most effective at reaching the poorest South Africans (Bassier et al., 2021).

The partial labour market recovery along with the roll-out of social assistance interventions did lead to some economic recovery for households. While the labour market shock was inequality enhancing— initially poorer households were worst affected and benefited least from the partial recovery— social assistance interventions were progressively targeted, with the lowest deciles of the populations benefiting disproportionately (Jain et al., 2020b; Köhler & Bhorat, 2020). Comparing incomes in April and June, Jain et al., (2020b) find evidence of a decrease in household poverty rates by between 3 and 6% points for the general population.

At the time of writing, most pandemic related restrictions have been reversed. The National State of Disaster was lifted on 5 April 2022, reflecting a shift in policy towards an acceptance that COVID-19 has entered an endemic state. Studies have revealed high levels of antibodies within the South African population, partly reflecting the achievements of the vaccination campaign (approximately 30 per cent of the population has been fully vaccinated), but also revealing the widespread circulation of the virus within the population (Madhi, 2022). The virus continues to circulate widely, though with low mortality rates.^2^ The R350 SRD grant, initially introduced for a period of six months, has been extended several times, most recently until April 2023. Despite an apparently robust labour market recovery following the initial pandemic shock, the South African labour market remains loose. The latest figures estimate the unemployment rate to be 35.3 per cent, the highest since Statistics South Africa began collecting quarterly labour force survey data in 2008 (Statistics South Africa, 2022).

## Data and methods

### Qualitative data

The main focus of this paper is on the analysis of two rounds of semi-structured phone interviews, conducted between June and September 2020. The 15 respondents, who are identical between rounds, were selected from a previous qualitative study conducted from July to September 2017 in Khayelitsha, a large African township situated about 30 km southeast of Cape Town’s city centre. Khayelitsha was selected as a study site because it closely resembles many of the context characteristics that typically condition the livelihoods of the urban poor in South Africa (Schotte, 2019; Zizzamia, 2020).^3^

Participants of the 2017 study were drawn from a sampling frame that had been designed to capture the local socio-economic diversity, covering different neighbourhoods and welfare levels (for details, see: Schotte 2019; Zizzamia, 2020). This previous study used a combination of focus group discussions (FGDs) and individual, in-depth life-history interviews (LHIs). Both research elements involved wealth ranking exercises: as part of the FDGs, four welfare levels—ranked from four (lowest) to one (highest)—were subjectively defined by participants within the local township context. The LHIs traced fluctuations in well-being on this four-point scale over respondents’ lifetime, and linked these fluctuations to their determinants.

For the present extension study, the LHI respondents were recontacted in early June 2020. Out of 31 original respondents, 11 could not be reached, one was deceased, five refused to be re-interviewed and 14 agreed to participate in this research. It is not possible to determine the reasons for the attrition of the 11 sample members who could not be tracked, and therefore whether this attrition is differential and might affect our analysis. For instance, it is possible that attrition rates are higher among those who migrated between 2017 and 2020, including migration as a response to the pandemic shock (Ginsburg et al., 2022; Posel & Casale, 2021). At the same time, in a context in which cell phone theft and loss are high and in which cell phones are often shared, it was to be expected that a considerable proportion of respondents would not be reached on the numbers that they provided three years prior. To improve the representation of the young population in Khayelitsha and increase our sample size, one additional respondent—a young male—was added from the 2017 sampling frame.^4^

**Fig. 2:**
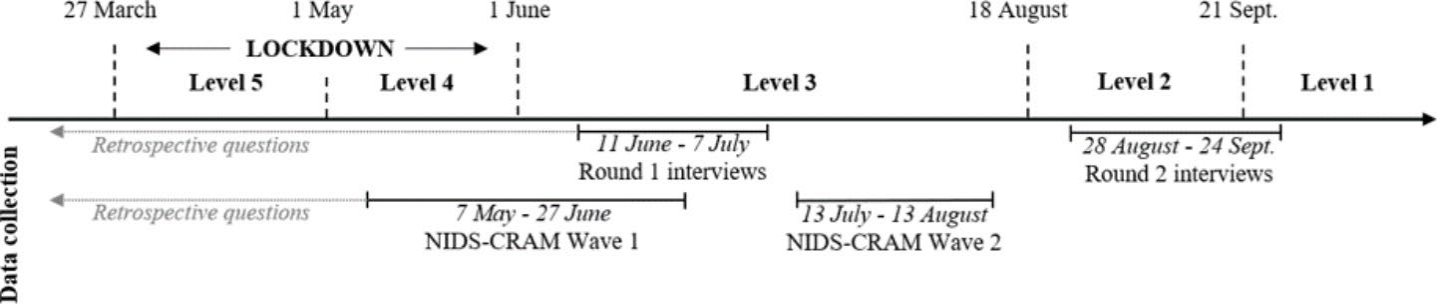
Timeline. (Source: authors’ illustration)

Figure 2 illustrates the timing of the data collection in the extension study. The first round of interviews was conducted from 11 June to 7 July 2020 (alert level 3), and the second round from 28 August to 24 September 2020 (alert levels 2/1).^5^ The first interview round included a set of retrospective questions to establish how the participants’ overall life circumstances had evolved between our last visit in 2017 and February 2020, before the pandemic had reached South Africa. The remainder of the interviews focused on how the participants’ situation had changed since the onset of the pandemic up to the time of the interview, including probing questions regarding their household’s ability to buy essential goods, changes in their own and close family members’ employment situation, and the schooling situation of children in the household. Respondents were also asked about their opinions regarding the implemented government response measures and, in the second round, the relaxation of the same. Unlike in 2017, the two interviews in 2020 did not include a structured household roster, and as a result we cannot systematically observe changes in household composition unless respondents explicitly discussed such changes.

As part of the interviews, respondents were asked to rank their welfare levels in February, June, and September 2020, using the same four-level welfare scale originally established in 2017. This allowed us to identify shifts in welfare—both in terms of the initial shock as well as the subsequent recovery—and relate these to the COVID-19 economic shock. Each interview round was coded and analysed using a thematic approach, following a similar methodology as proposed by Nyashanu et al., (2020).^6^

To supplement the evidence gathered, two key informant interviews were conducted after participant interviews had been completed. The two key informants were leaders of two different non-governmental community-based organisations, both of which were involved in providing support to households during the pandemic shock and coordinating support from within and outside the community. Both key informants were selected for the insights they could give on the effect of the pandemic on local communities and households, and the role played by state and community actors in dealing with the shock. These were designed to provide background information on broader changes in the township environment attributable to the pandemic, which were brought up as relevant by some of the interviewees.

### Quantitative data

Preceding the qualitative analysis, to contextualize individual accounts and provide a broader perspective, we present a snapshot of the nationwide dynamics observed using quantitative data. The panel data are derived from the National Income Dynamics Study: Coronavirus Rapid Mobile Survey (NIDSCRAM) (NIDS-CRAM, 2020a, b) as well as pre-pandemic waves of NIDS (NIDS, 2017).^7^

The NIDS-CRAM study has facilitated reliable assessments of the economic, health, and social impacts of COVID-19. The panel dimension allows assessing how various outcomes have changed over the course of 2020, using a combination of repeated interview rounds and retrospective questions. There are five waves of NIDS-CRAM data available that were collected over the course of May 2020 to July 2021. Our analysis focuses on the first two NIDS-CRAM waves, for which the timing coincides with our qualitative data collection (see Fig. 2 above). The first wave was administered between 7 May and 27 June and asked retrospective questions about February (pre-lockdown), April (alert level 5), and the last seven days (alert levels 4/3). The second wave was administered between 13 July and 13 August 2020 (alert level 3), including retrospective questions about June (alert level 3).

To draw conclusions about how pre-pandemic economic conditions shaped the vulnerability of households to the pandemic shock, we merge data from NIDS-CRAM with NIDS 2017. The first wave of NIDS-CRAM provides data on 7,074 respondents drawn from the adult sub-sample of NIDS 2017.^8^ Out of these, we could classify the pre-pandemic poverty status of 7,061 respondents with available expenditure information in 2017. This is the main sample used in the quantitative analysis of this paper. In the second wave of NIDS-CRAM, 5,676 respondents were successfully reinterviewed, out of which we could classify the pre-pandemic poverty status of 5,666 respondents. We use this sample in the last part of the quantitative analysis to assess signs of recovery between the first and second half of 2020.

## The economic impact of the pandemic: a quantitative snapshot

This section presents evidence on the immediate economic impact of the COVID-19 pandemic on households in South Africa. First, we assess the magnitude of the initial shock to household income and discuss potential implications for poverty and food insecurity. Second, we investigate heterogeneity in the experience of the shock and link this assessment to pre-existing markers of vulnerability. Third, we provide evidence on the extent of economic recovery in the early post-lockdown period.

### Immediate shock of the COVID-19 pandemic

**Fig. 3:**
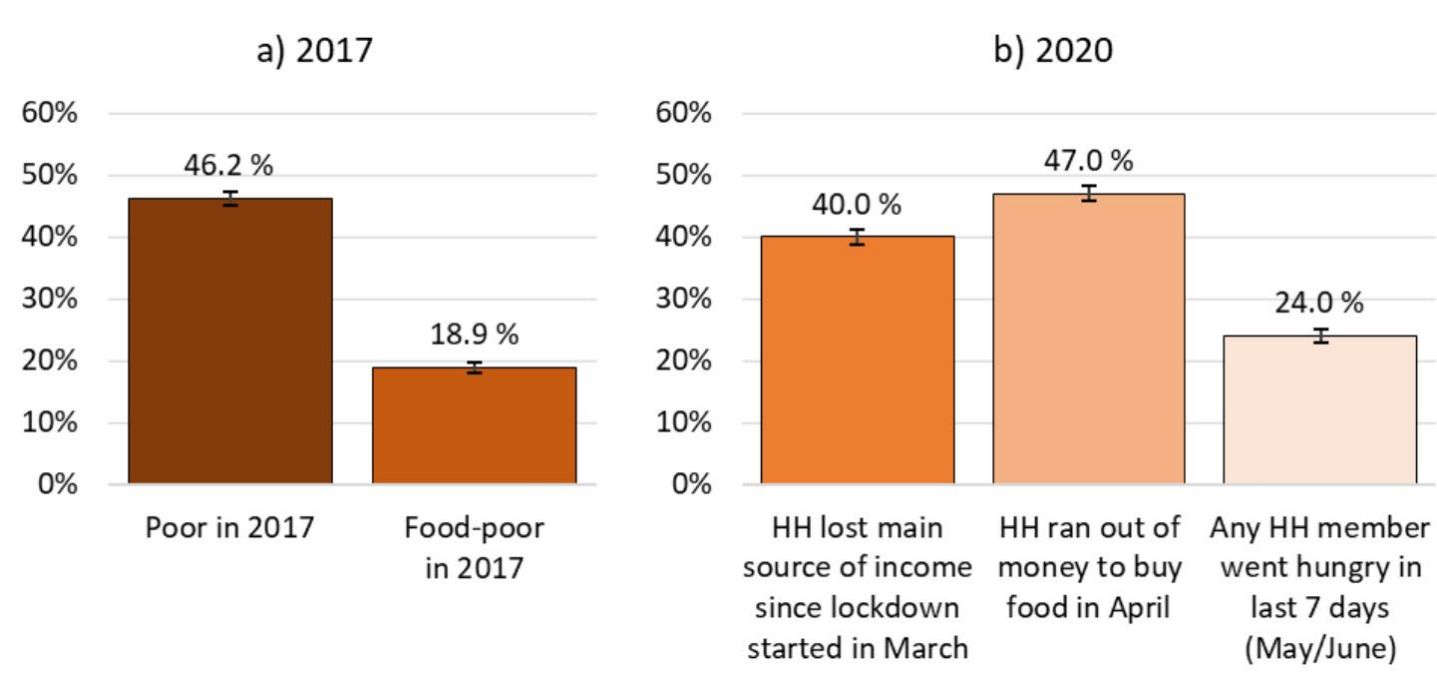
Event prevalence. (Note: estimates for weighted NIDS-CRAM adult population with 95% confidence intervals. Poverty status in 2017 is defined based on household per capita expenditure in relation to national upper-bound and food poverty lines. HH abbreviates household. Source: authors’ compilation based on NIDS wave 5 and NIDS-CRAM wave 1)

In 2017, 46 per cent of NIDS-CRAM respondents were poor by national standards. That is, they were lacking the financial means to cover basic needs. Moreover, 19 per cent were food-insecure. That is, their household would have been unable to purchase sufficient food to fulfil caloric requirements, even if all expenditure was dedicated to food (Fig. 3a).^9^

Figure 3b presents three indicators of economic distress experienced in the early phases of the pandemic. Firstly, 40 per cent of NIDS-CRAM respondents reported that their household had lost its main source of income between the start of the lockdown on 27 March and April 2020.^10^ Secondly, 47 per cent of respondents said that their household ran out of money to buy food in the month of April. This presents a substantial rise compared to pre-COVID outcomes. According to estimates by van der Berg et al., (2020) drawing on data from the General Household Survey (GHS),^11^ back in 2018 a much smaller share of 25 per cent reported running out of money for food at any point in the past year, a far less demanding criterion. The experience of running out of money to buy food is likely conditioned by usual consumption patterns. While it signals severe financial pressure, it may not always translate into food insecurity (i.e. ‘going hungry’). This is, households may still be able to find ways to put food on the table, for example, by opting for less expensive foods, through support provided by social networks or (non-)government programmes, drawing down savings, or borrowing (van der Berg et al., 2020). Nonetheless, as the third indicator shows, 24 per cent reported that at least one household member went hungry in May or June 2020. While not directly comparable to the expenditure-based measures of food poverty presented in Fig. 3a, this points to a likely rise in the incidence of food insecurity in the early phases of the pandemic.

### Vulnerability factors and heterogeneity in the shock experience

**Fig. 4:**
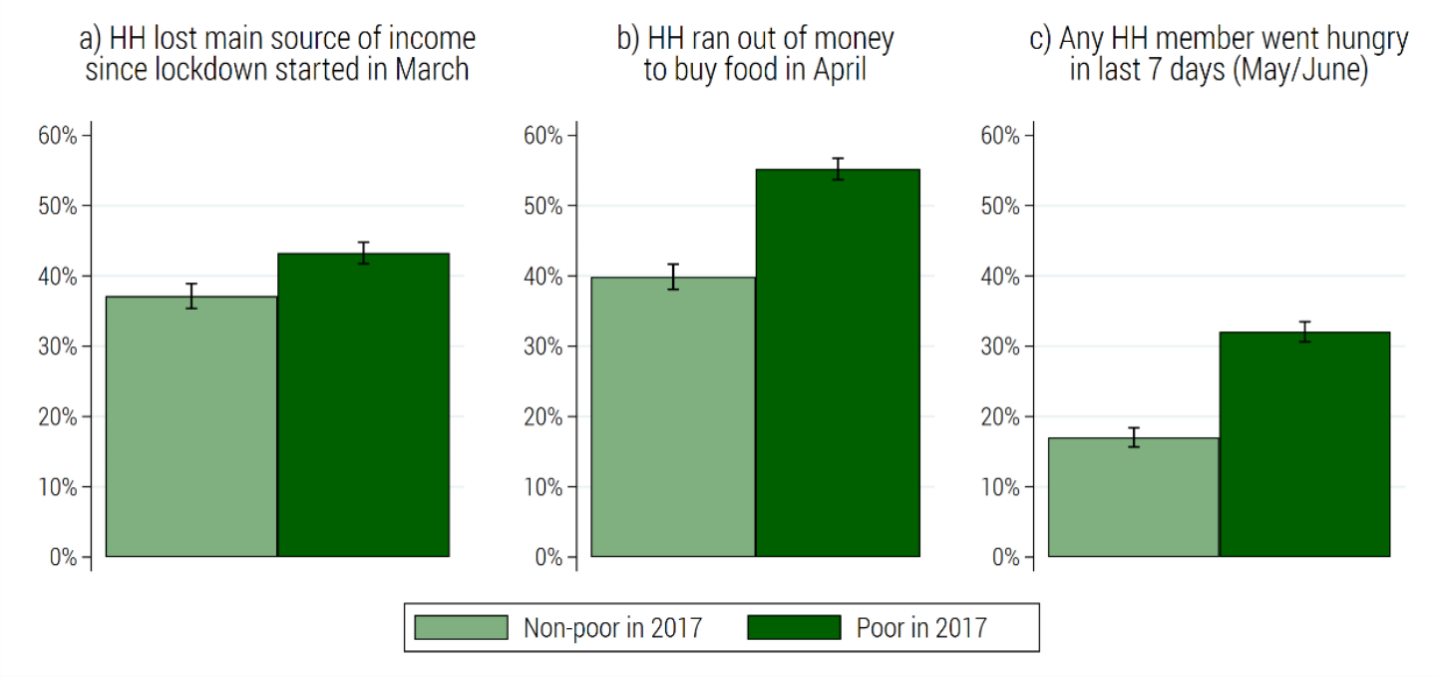
Event prevalence by poverty status in 2017. (Note: estimates for weighted NIDS-CRAM adult population with 95% confidence intervals. Poverty status in 2017 is defined based on household per capita expenditure in relation to national upper-bound and food poverty lines. HH abbreviates household. Source: authors’ compilation based on NIDS wave 5 and NIDS-CRAM wave 1)

Respondents who had been poor in 2017 were more likely to report economic distress in 2020 (see Fig. 4). This is expected, as households with insufficient means to cover basic needs are often unable to build up a financial cushion to buffer economic shocks. However, as Fig. 4 shows, a substantial share of respondents who had been non-poor in 2017 was also vulnerable to the pandemic shock.

Given the patterns observed in Fig. 4; Table 1 explores discrepancies in the incidence of economic distress experienced prior- and post-COVID-19 by different population groups. In 2017, the incidence of poverty, especially food poverty, was substantially higher among respondents in rural compared to urban areas. This geographic gap is remarkably less pronounced in the outcome measures for 2020. Importantly, respondents living in informal housing, concentrated in urban peripheral areas, showed the highest incidence of financial distress since the start of the lockdown—with 50 per cent reporting losing their main source of income, 65 per cent running out of money for food, and 36 per cent going hungry. Moreover, while labour earnings and remittances shielded respondents against poverty in 2017, households relying on these income sources were at a high risk of losing this source of income and running out of money to buy food during the lockdown (see Table 1).

The profiles of those who came under economic distress since the onset the COVID-19 pandemic thus differ in some respects from those who had experienced poverty previously, as shown in Table 1. They are considerably more likely to be located in informal urban settlements, and more reliant on labour earnings as their main source of income. However, we may expect that many of those who newly experienced financial distress in 2020 had previously been on the brink of poverty. That is, despite being able to cover basic needs in 2017, they faced a higher risk of falling into poverty in the event of economic shocks.

**Table 1:** Event prevalence by individual characteristics

		2017		2020	
	**HH was** **poor**	**HH was** **food-poor**	**HH lost main source of income since lockdown started in March**	**HH ran out of money to buy food in** **April**	**Any HH member went hungry in last 7 days (May/June)**
**Total**	46.2%	18.9%	40.0%	47.0%	24.0%
	(0.6)	(0.5)	(0.6)	(0.6)	(0.5)
**By location**					
Rural	58.4%	28.2%	43.0%	51.7%	31.3%
	(1.2)	(1.1)	(1.3)	(1.3)	(1.2)
Urban	43.6%	16.9%	39.3%	46.0%	22.4%
	(0.7)	(0.5)	(0.7)	(0.7)	(0.6)
*Ratio rural/urban*	*1.3*	*1.7*	*1.1*	*1.1*	*1.4*
	*(0.06)*	*(0.12)*	*(0.06)*	*(0.05)*	*(0.10)*
**By housing type**					
A house or flat	42.0%	15.7%	38.5%	44.4%	21.1%
	(0.7)	(0.5)	(0.7)	(0.7)	(0.6)
A traditional house	75.1%	45.4%	43.2%	51.4%	35.6%
	(1.5)	(1.7)	(1.7)	(1.7)	(1.7)
An informal house	56.9%	22.3%	50.4%	65.1%	36.2%
	(1.9)	(1.6)	(2.0)	(1.9)	(1.9)
*Ratio traditional/informal*	*1.3*	*2.0*	*0.9*	*0.8*	*1.0*
	*(0.08)*	*(0.27)*	*(0.08)*	*(0.05)*	*(0.12)*
**By main income source**					
Labour	35.5%	11.9%	43.5%	40.4%	17.7%
	(0.8)	(0.6)	(0.9)	(0.9)	(0.7)
Government grant	64.2%	30.4%	33.4%	56.1%	31.5%
	(0.9)	(0.9)	(0.9)	(1.0)	(0.9)
Remittances	46.0%	17.2%	47.0%	55.0%	32.8%
	(2.3)	(1.7)	(2.3)	(2.3)	(2.2)
Other	27.9%	9.0%	27.9%	28.1%	15.9%
	(4.0)	(2.6)	(4.1)	(4.1)	(3.3)
*Ratio grants/labour*	*1.8*	*2.6*	*0.8*	*1.4*	*1.8*
	*(0.07)*	*(0.19)*	*(0.04)*	*(0.07)*	*(0.13)*
*Ratio grants/remittances*	*1.4*	*1.8*	*0.7*	*1.0*	*1.0*
	*(0.10)*	*(0.26)*	*(0.06)*	*(0.07)*	*(0.11)*
**Number of observations**	7,061	7,061	6,894	7,007	7,010

Note: estimates for weighted NIDS-CRAM adult population. Standard errors in parenthesis. Standard errors of the ratios have been bootstrapped with 100 replications. HH abbreviates household

Source: authors’ calculations using NIDS wave 5 and NIDS-CRAM wave 1

It should be noted that the results reported in Table 1 do not account for potential migratory responses to the pandemic shock. Ginsburg et al., (2022) and Posel & Casale (2021) demonstrate that in the South African context of spatially ‘stretched’ households and circular migration between rural (labour sending) and urban (labour receiving) areas, the pandemic shock led to substantial urban-to-rural migration. Posel & Casale (2021), using NIDS-CRAM data, estimate that 16 per cent of adults in South Africa moved households during the early months of the pandemic. This migratory response was likely a coping mechanism designed to offset negative income and labour market effects of the shock, implying that those who were economically worst affected by the pandemic were also most likely to migrate to rural areas. Similarly, some of those worst affected by the pandemic shock may have moved into informal housing from formal housing. In Table 1, we are not able to capture these dynamics, and the displayed patterns should be interpreted with these caveats in mind.

To investigate how pre-pandemic conditions affected the risk of falling into poverty in reaction to the COVID-19 shock, we need a measure of vulnerability that has more structural signal than previously realized expenditure levels alone. The availability of panel data spanning the pre- and post-COVID period provides a unique opportunity in this regard. It allows us to investigate the individual- and household-level characteristics that conditioned poverty entries and exits prior to the pandemic, and use these to assess the ex-ante vulnerability to poverty among NIDS-CRAM respondents. On this basis, we can divide the NIDS-CRAM sample into five social strata, using the multilayered stratification schema suggested by Schotte et al., (2018).^12^ The approach starts from a standard division of the sample into three main classes based on monetary thresholds: the poor, the middle class, and the elite.^13^ Among the poor, we then distinguish those with below-average chances of exiting poverty and thus a comparatively high ex-ante risk of poverty persistence—the chronic poor—from those with above-average chances of making it out of poverty—the transient poor. Analogously, among the middle class, we distinguish those who face an above-average ex-ante risk of slipping into poverty—the vulnerable—from the more economically stable and secure ‘true’ middle class. The poverty risk scores underlying this classification are calculated based on pre-COVID characteristics recorded in 2017.

**Fig. 5:**
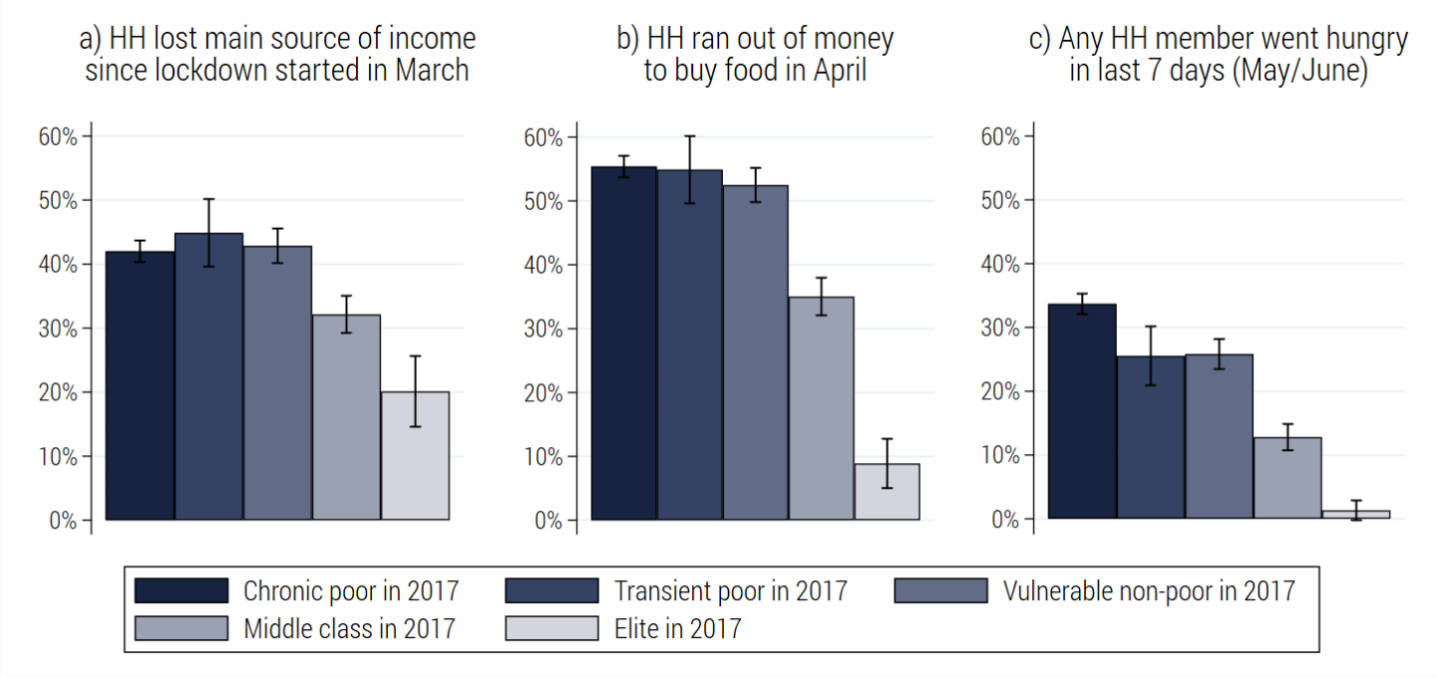
Event prevalence by economic class in 2017. (Note: estimates for weighted NIDS-CRAM adult population with 95% confidence intervals. Class categories based on Schotte et al., (2018) and Zizzamia et al. (2019). HH abbreviates household. Source: authors’ compilation based on NIDS wave 5 and NIDS-CRAM wave 1)

Figure 5 illustrates the results. We find that economic distress since the onset of the COVID-19 pandemic was experienced by respondents across the income range. However, with respect to all three indicators, the incidence is significantly lower among those who had previously been considered as stably middle class or elite. In contrast, the transient poor and the vulnerable non-poor faced the highest risk of job loss, were similarly exposed to severe financial pressures as the chronic poor, and experienced elevated levels of food insecurity. Analysing the pandemic impact, the pre-pandemic position in the labour market may provide an important indication. Respondents who were more resilient to the shock (i.e. the middle class and elite) were more likely to be formally employed ex-ante, with a permanent work contract and union coverage. By contrast, the transient poor and the vulnerable were more likely to be in unstable and informal employment relationships, and a larger share was either unemployed or economically inactive prior to the pandemic (Schotte et al., 2018; Zizzamia et al. 2019).

Our findings align with other studies identifying precarious forms of work as key indicators of preexisting vulnerabilities that materialized during the COVID-19 crisis. For example, using NIDS-CRAM data, Espi et al., (2020), Jain et al., (2020b), and Ranchhod & Daniels (2020a) show that job losses were more severe for those in the informal sector, for unregulated workers within the formal sector, and for those with a historically weak attachment to the labour market. These job losses often resulted in a descent into poverty (Jain et al., 2020a). Similar findings were obtained by studies conducted in other sub-Saharan African countries (see, inter alia, Lakuma & Nathan 2020, Balde et al., 2020, Schotte et al., 2021).

### Early signs of recovery from the shock

**Fig. 6:**
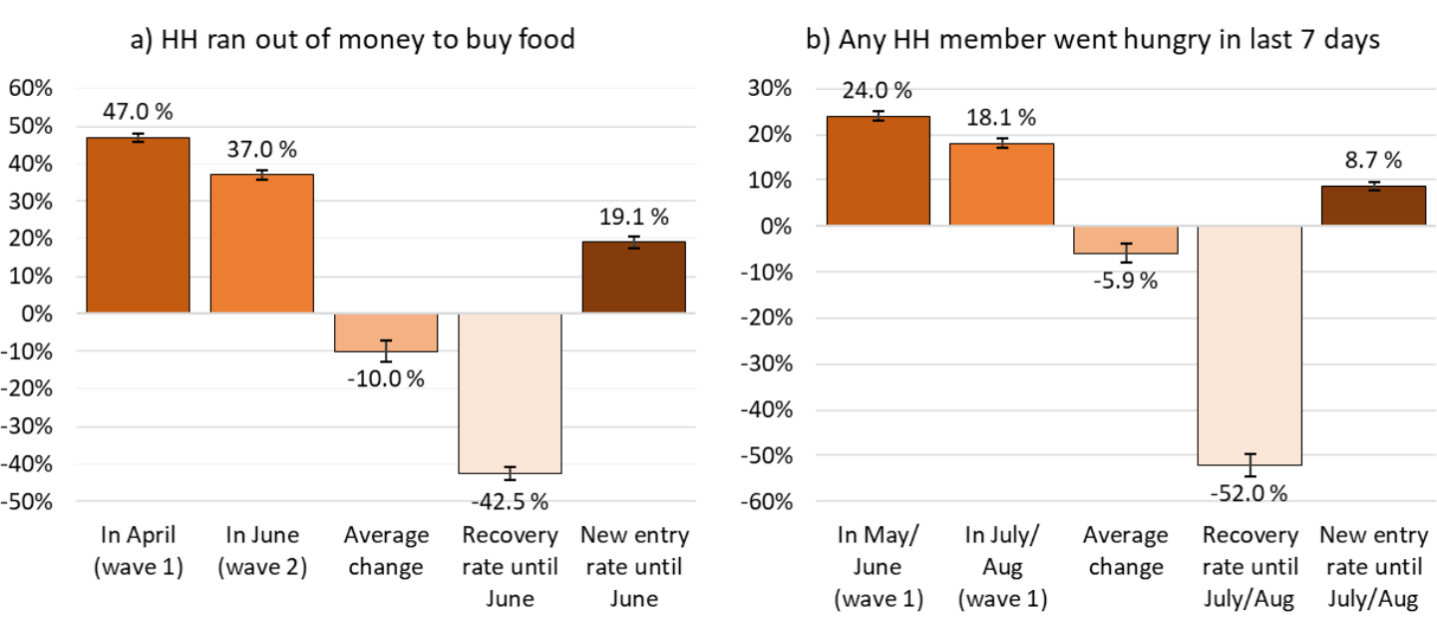
Changes in event prevalence. (Note: estimates for weighted NIDS-CRAM adult population with 95% confidence intervals. HH abbreviates household. Source: authors’ compilation based on NIDS-CRAM wave 1 and wave 2)

Figure 6 gives an indication of the extent to which economic pressures on South African households have eased since the most rigid lockdown restrictions were lifted and grant relief measures came into effect (see Sect. 2). Between April and June 2020, the average share running out of money to buy food dropped by 10% points. Mirroring these patterns, the incidence of hunger was 6% points lower in July/August compared to May/June 2020 (Fig. 6b).

Importantly, it was not necessarily the same respondents who reported experiencing these events. Out of those who had run out of money to buy food in April, 43 per cent said they were able to cover their food expenditures in June. This may be attributable to a rise in available economic resources, but could also be explained by adjustments in consumption patterns or support received through social networks. However, out of those who had been able to cover their food needs in April, 19 per cent reported running out of money for food in June (Fig. 6a). Similar dynamics underlie net changes in hunger: 52 per cent of households where at least one member had gone hungry in May/June did not report hunger in July/August. At the same time, nine per cent of respondents who had not experienced hunger in the household in May/June reported hunger in July/August.

These findings indicate a moderate recovery in household economic welfare in the second half of 2020. However, our findings also suggest that some households that appeared to be able to buffer the immediate shock had subsequently succumbed to the economic pressure in later periods. Both these observations resonate with the findings by other studies using NIDS-CRAM data (Jain et al., 2020b; Köhler & Bhorat, 2020; Ranchhod & Daniels, 2020b).

## The impact of the pandemic on livelihoods: a deep qualitative assessment

**Fig. 7:**
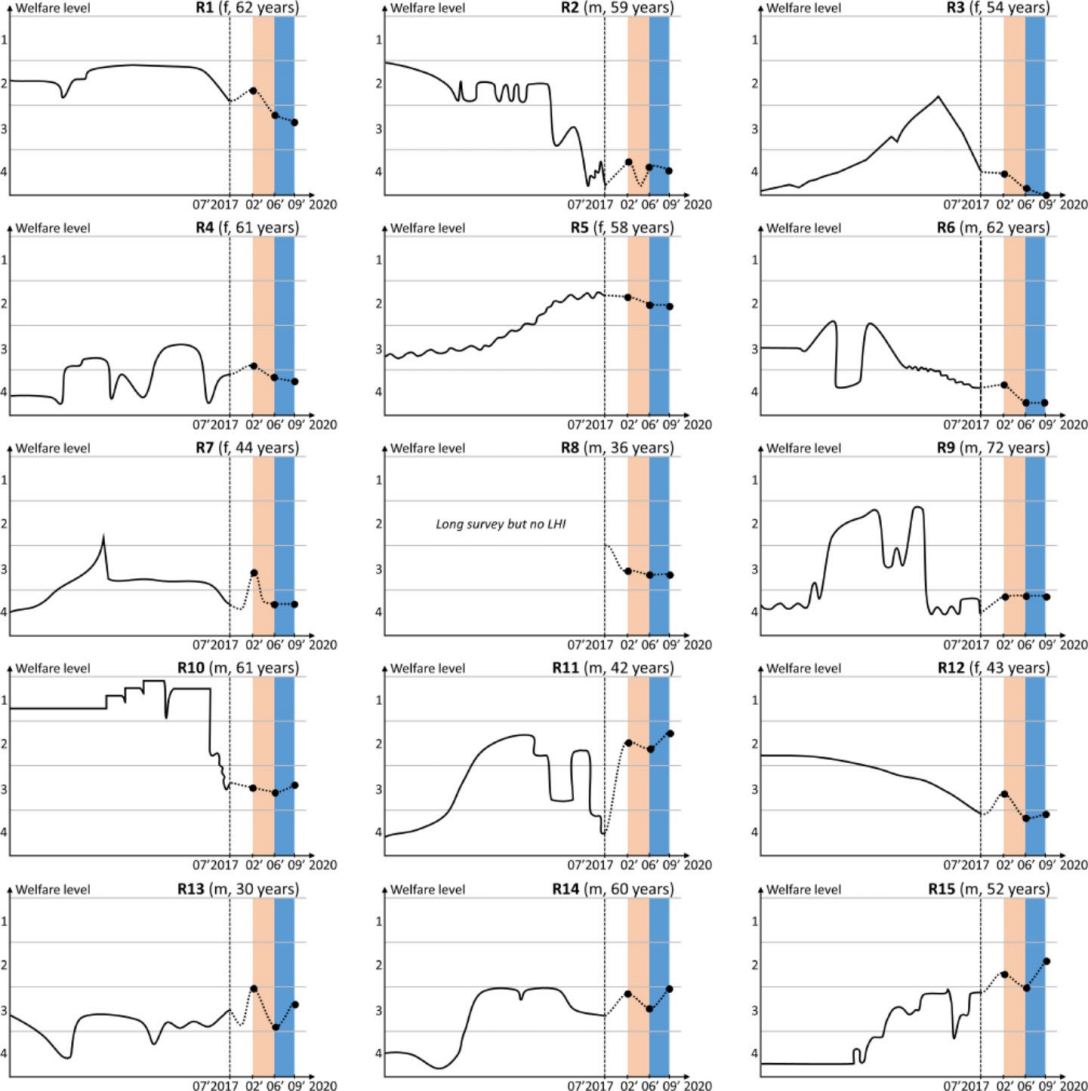
Patterns of livelihood dynamics. (Note: respondents were assigned numbers to anonymize data. R = respondent, f = female, m = male. The two shaded areas respectively indicate the first and second round of interviews conducted in 2020. Source: authors’ graphical presentation based on qualitative research data)

The COVID-19 pandemic and related policy measures—particularly through the immediate shock to labour markets—had important implications for household welfare. Our qualitative evidence confirms this. The life history diagrams presented in Fig. 7 give an overview of the perceived livelihood dynamics reported by respondents (see Appendix Table A.1 for details on respondent characteristics).

During the original study conducted in 2017, we asked participants to recount their individual life history, starting from their parental background and living conditions during childhood, up to and including the present. As visual aids, all events reported during the interview were recorded on two sets of cards, where one colour was assigned to positive events and another colour to negative events. At the end of each interview, respondents were asked to rank their own welfare level at different points in time on a four-point scale.^14^ With the help of the interviewee, the researcher would then map out the respondent’s life trajectory on a large sheet of blank paper (in line with the methodology suggested by Davis & Baulch 2011), capturing the respondent’s welfare level at each stage in life and the events that had caused transitions within and between welfare categories.

The interviews in this extension study focused on the impact of the pandemic on economic livelihoods and well-being. Respondents were asked to rank their welfare in February, June, and September 2020 in relation to their situation at our last visit in 2017, using the same four-point scale.

Out of 15 participants, 14 reported a decline in household welfare between February and June 2020 (Fig. 7). Among these 14, only one (R5) saw no change in labour earnings (being a public school teacher) but instead reported a fall in rental income as her tenant had lost her job and left the city. In the remaining 13 cases, the decline in labour income was experienced either by respondents themselves, a household member, or a family member who had been supporting the household financially. Almost all (11/13) explicitly identified this negative labour market event as the driver of downward mobility between February and June. In the only case where no decline was experienced (R9), both household members were elderly and relied exclusively on the old-age pension grant.

The patterns observed during the second study period from June to August 2020 are much more mixed: 4/15 reported a continued but attenuated deterioration in welfare (R1–R4), 5/15 a stabilisation (R5–R9), and 6/15 saw a full or partial recovery (R10–R15) (see Fig. 7). This recovery was mainly facilitated by respondents being able to return to work, as discussed below.

Interestingly, we find no strong connection between the respondents’ pre-COVID welfare trajectories, and the magnitude of the initial COVID-shock and near-term recovery. That is, respondents who experienced higher volatility or downward mobility over their life course were not consistently more vulnerable to the crisis. However, it is important to note that by focusing on township residents, we are looking at an economically vulnerable segment of the population. The vast majority of respondents were either poor or vulnerable to poverty back in 2017—both in their own perception (approximated by levels 3 and 4 in Fig. 7) and evaluated based on reported household characteristics (approximated by PPI in Appendix Table A.1). Revealing an important extent of heterogeneity in this vulnerable segment, the extent of the pandemic shock mainly depended on the respondent’s economic situation just before the crisis, especially with regard to the sources of household income, attachment to the labour market, number of dependents, and the existence of savings or other assets to buffer economic losses.

### Economic losses in the labour market

Overall, in our sample, the labour market shock affected a population which does not have a particularly strong attachment to the formal labour market, but who nevertheless remain heavily reliant on labour income—often derived from labour income shared within extended support networks and from informal work. In line with the quantitative evidence (Jain et al., 2020b), we find that a more robust labour market recovery was experienced by those who had maintained an active employment relationship over the lockdown—especially if this employment relationship was formal. However, as formal sector businesses were also affected by government regulations and the overall drop in demand, even formal jobs were not necessarily secure and, in instances, experienced a partial informalization.

Out of four respondents (R5, R11, R13, R15) who had been formally employed prior to the pandemic, only one (R5) saw no change in labour earnings. As a government-employed school teacher, her salary remained unaffected. Another respondent (R11), a supermarket worker, continued working throughout the lockdown. Nonetheless, he experienced a decline in earnings due to a reduction in hours worked. The other two respondents were on unpaid leave during the lockdown. Only one of them (R15) received UIF payments (after substantial delays), while the other was ineligible because of insufficient tenure. By September, both had resumed work.

Even though all four formal wage employees were able to resume work in the post-lockdown period, the pandemic did not leave these jobs unaffected. For example, R15 explained that the company he was working for was experiencing severe financial difficulties since the onset of the pandemic and stated: “I am noticing that after this coronavirus things are not stable [at the firm]. Even the bosses look weak now because there are rumors that the company may be closed.” He also reported an informalization of his previously formal employment relationship. Talking about himself and his coworkers, he said: “We have just been de-registered from the Building Industry Bargaining Council and there won’t be any deductions now. You will be given your money and save it yourself. That is what worries me now.”^15^ Increased job instability left workers more vulnerable to future shocks (see Sect. 5.2) and contributed an overall feeling of insecurity and consequent psychological distress (see Sect. 5.3).

Those in informal work were especially vulnerable to the labour market shock. Among the two respondents (R7, R12) who had been in informal wage employment prior to the pandemic, R12 was able to continue working at reduced hours during the lockdown. By September, she was still working a reduced number of days. The other, R7, had been laid off during the lockdown. Moreover, all four respondents (R2, R4, R8, R10) who had been running informal enterprises prior to the pandemic had either terminated or scaled back their activity by June, and only one (R10) had resumed operating at pre-lockdown capacity by September. Respondents mentioned three main reasons to explain this break in business activity: lockdown regulations, challenges in transportation and in procuring stock, and a fall in demand. Regarding the latter, one of our key informants emphasized the interdependence between formal and informal sector activities, arguing that informal businesses rely on the spending of those with incomes from the formal economy—using his example: selling snacks on trains or at stations is not possible if nobody is going to work. A recovery in the informal economy will thus depend on a prior recovery in the formal labour market.

The shock to the labour market was also felt by the unemployed and those outside the labour force. Four non-working sample members (R1, R3, R6, R14) reported in June that they no longer received the same support from family members because their benefactors had lost jobs or earnings. This highlights the importance of labour income in sustaining much broader networks than a worker’s immediate household, and the vulnerability of this mechanism of support and insurance to large, covariate labour market shocks (see Sect. 5.2).

### Amplified vulnerabilities, risk factors, and resilience

In addition to the labour market shock, our qualitative interviews highlight two additional dimensions of amplified vulnerabilities and emerging risk factors. First, households with limited assets to withstand a sudden economic loss responded to the crisis by running down savings and defaulting on insurance payments, leaving them yet more vulnerable to future economic shocks. Second, school closures posed a double burden to children from socio-economically disadvantaged backgrounds. The absence of meals provided at schools posed risks exacerbating food insecurity; and many were lacking the basic infrastructure to continue remote learning, reducing their chances of educational attainment and future upward social mobility.

#### Social security mechanisms

The success with which households were able to withstand the pandemic shock depended largely on their ability to access formal or informal systems of social protection.

In face of the COVID-19 labour market shock, government grants provided an essential, stable stream of income. At least 11/15 interviewees reported living in households with access to grant income. For these households, the top-up to government grants, issued from May 2020 onward, provided some buffer to the negative income shocks they experienced. In many cases, respondents and their households relied primarily or even exclusively on social grants when labour incomes collapsed, and would have been left destitute in their absence. While in most cases the grant income was used to cover immediate consumption needs, we also found evidence of social grants being used as strategies for accumulation and insurance. In several cases (R2, R4, R9, R14), grants were used to invest in durable assets (like housing repairs or improvements) or as start-up capital for survivalist enterprises once the economy had more fully reopened in September. However, many also complained that the top up was insufficient, given the economic challenges they faced, including rising prices for basic items. For example, one old-age pension recipient (R1) said: “Things have gone from bad to worse because I survive only on this social grant with three other people depending on it. My children have not received jobs yet.”

In addition to public social welfare schemes, informal insurance mechanisms can provide protection against the impact of economic shocks and earnings volatility. While the COVID-19 pandemic has delivered such a shock, it undermined at the same time the present and future effectiveness of these mechanisms. A strong example of this effect was given by one respondent (R11), who (together with his wife) had been contributing to a stokvel—a rotating savings and credit association—prior to the pandemic. R11 was worried that his household or other members of the group would fail to pay their contributions, saying: “Now we are not sure whether to continue [contributing] because of the current situation. There are [other stokvel members] who work at a coffee shop […] so they stopped working during the lockdown. […] So it is going to be difficult to fork out ZAR1,500 [semi-annual contribution].” This account reveals how informal financial instruments are often effective for managing idiosyncratic risks—affecting individuals or groups of individuals—while being less effective at dealing with large covariate shocks—simultaneously affecting entire communities (Dercon, 2002). Our qualitative data provides insight into this process, which is not well captured in the quantitative data.^16^ Several respondents (R1, R3, R4, R14) reported being able to call on family members in a time of crisis, but that this was seen as an option “of last resort” (R1) since these family members also supported others and were negatively affected by the pandemic themselves. Similarly, typical patterns of mutual support between neighbours were affected by the pandemic.

To buffer the loss of household income, several respondents were forced to run down savings and/or to default on insurance payments, leaving them vulnerable to future shocks—including the health risks posed by the pandemic. For example, one respondent (R10) said: “Economically and health-wise I am worried because if anything would happen I don’t know where I would go or where to start. […] Like if any of my family members were to die I am not sure how I would bury them because I am not working and my policies lapsed.” In this case, the relative stability in observable living standards (see Fig. 7) masks the increase in underlying economic vulnerability. Cutting back on savings and insurance to meet basic needs in the present may risk potential ruin in the future. Moreover, it may also block avenues of social upward mobility, as the example of a young male respondent (R13) illustrates. Before the pandemic hit and he was temporarily laid-off from work, he had been saving money to acquire a certificate that would enable him to work as a petrol attendant. Now that his financial situation had changed, he was no longer able to contribute to the stokvel that he had joined with the aim of using the payout to finance his training. As people recover economically, they will have to catch up on insurance installments or face the risk of remaining vulnerable. The former choice would hold back the pace of the economic recovery, while the latter would increase vulnerability enduringly.

#### School closures

The pandemic may have lasting implications for children’s development and future prospects of social upward mobility. Reduced food consumption coupled with school closures and the constraints that poor children face in online teaching may have a negative effect on human capital formation, with potentially lasting consequences.

Overall, in our sample, the economic shock of the pandemic affected a population with high ex-ante vulnerability, leaving many prone to food poverty. During the June period, 8/15 respondents reported that they had cut back on food expenditure and had resorted to reducing the quantity and/or variety of food consumption. Moreover, among the respondents with children in the household, 5/12 reported additional pressure on household budgets due to children losing out on school feeding programmes during the lockdown.

In addition, the closure of schools and universities caused major disruptions to students’ learning. One of our respondents (R5), who is a primary school teacher, explained that schools were often unable to contact parents during the lockdown, and many children had been left behind during the period of homeschooling: “Some of them were helped by parents, but others were just left on their own. […] There are those [who] have the potential to pass but I don’t want to lie, many of them are struggling and will surely repeat this year.” This general concern about failing the school year was echoed by other respondents in the sample, expressing concerns about their children being left with an insecure future.

Given that respondents in our sample typically do not own computers or tablets, having a smartphone with internet access appeared to be a key determinant of whether or not schooling could continue. While some respondents reported that their children had received school exercises (R12) and university assignments (R9) on their phones, the majority said they had not received anything. For instance, the daughter of one respondent (R7), whose phone was not equipped to receive any exercises, reported feeling disadvantaged compared to her peers who had better phones and had received the tasks. She also did not feel supported by teachers in catching up with the material when schools reopened, and reported that teachers were running through material too quickly, trying to make up for lost time. She described the situation as “learning in a pressure situation”, which caused her to feel overwhelmed and—despite having passed the trial exams in March—left her without hope of passing her upcoming final school-leaving exams. Differences in the ability to access remote learning may exacerbate existing educational inequalities, with the children most in need of close attention belonging to those households which could not be contacted and which did not have resources to pursue remote learning under parental supervision.

### Psychological distress in a context of vulnerability and uncertainty

Over the course of our interviews, one respondent (R5) reported testing positive for COVID-19. The health impact on her and her family (who were also infected) was mild. Among the other respondents, the health consequences of the pandemic were rather reflected predominantly in elevated levels of psychological distress.

The relationship between the pandemic shock and mental health is being studied in South Africa using NIDS-CRAM data.^17^ For example, Shepherd (2022) has shown that the increase in food insecurity is likely responsible for some of the increase in depressive symptoms observed in South Africa, while Posel et al., (2021) establish a plausibly causal link between job-loss and an increase in depressive symptoms. We add to this discussion by bringing qualitative data to bear on the effects of the pandemic on mental health.

Drawing on our interviews, we suggest that the psychological distress we observe among our interlocutors can be usefully understood as a perceived loss of individual agency and control. The corollary is an increase in a fatalistic sense that the pandemic’s unpredictable momentum came to play the predominant role in determining individual life outcomes in a context of vulnerability and uncertainty. This sense relates to both the perceived inability to determine one’s own health outcomes in a pandemic context, as well as the perceived inability to secure viable livelihood strategies. A key informant described this as a perceptible change in the overall mood, saying: “it’s a big thing […] when you can’t imagine how things will improve.”

Confusion, uncertainty, and a loss of individual control dominated the overall sentiment—especially during the first round of interviews. Respondents were sceptical about the government response measures, but largely expressed compliance with these, giving the state the benefit of the doubt given the lack of alternatives. One respondent (R7) summed up the general attitude, stating: “I am not sure about the truthfulness or safety of these measures, but we [comply] because we are told to.”

Within this general context, some pertinent differences in concern were observed across demographic groups: older women and those with family were more worried about the health risks posed by the pandemic and placed more emphasis on complying with hygiene and social distancing regulations. They also expressed concern about people ignoring the rules—reflecting an attempt to maintain some control over their environment. One elderly female respondent (R4), who used to supplement her pension by selling grilled intestines, reported stopping her business because of being “terrified” about catching the virus. Another respondent (R11) expressed his concerns about the risk of infecting his family and the limited actions he could take to prevent this, as he continued his work at a grocery store throughout the lockdown: “The shop is always packed […] so I meet these different people and come back home. […] It is even more difficult for us people living in hokkies [small shacks] because we are in the same room and there is no way I can isolate myself from them.”

Young males were much less worried about the health risks in the pandemic context. For instance, one young man (R13) admitted that “last weekend I went out to drink […] and there were […] seven of us in a hokkie. We […] were not wearing masks or any protective gear […]. We make a joke about it when we were drinking and someone coughs.” In the second round of interviews, the same individual tellingly explained that “I have always seen [the virus] as something far from me”. While expressing little concern about the health risks, young men expressed much more concern with the uncertainty surrounding the labour market, reflecting where they have ‘skin in the game’. The general uncertainty and sense of individual impotence “stressed” the young men in our sample (R13), battling with the perception that “there is nothing that can be done now” (R8). These findings align with cross-country evidence suggesting that young people are more affected by the mental health consequences of the pandemic than older people, and that these negative psychological effects are largely caused by stress and financial uncertainty (Varna et al., 2021).

Another aspect, which has been a global matter of concern during the pandemic, regards the rise in domestic violence, predominantly against women. Stay-at-home orders, unemployment, heightening economic pressures, and psychological distress tend to intensify existing tensions. One of our female respondents (R12) reported that the cut in earnings due to the lockdown created tensions in the household, and fights with her husband escalated more as during the lockdown they “were both in the house”. While she denied experiencing physical violence from her husband, the psychological harm was salient and dominated her story as well as her assessment of her own overall situation.

By September, the tone of interviews had shifted from the acute distress and uncertainty of the first round to a more resigned and passive tone. Although there was substantial heterogeneity among respondents, most supported the progressive relaxations in social distancing regulations, emphasizing the toll these measures had taken on livelihoods within their communities. Some were emboldened by the perception that the virus had not proved as devastating in terms of community health as was initially feared. For instance, one respondent (R6) claimed that “[it] gives us hope [that] even though it’s something huge, we have not buried someone because of this virus.” While health concerns were less acute stressors in the second round, the majority of respondents remained concerned about their economic situation. Especially those who had not been able to return to work expressed a general sense of stagnation and frustration (e.g., R6 saying: “There have been no improvement because I am still at home. Nothing is happening, so it’s still the same.”). Grant recipients also felt increasingly under pressure, as unemployed family members continued relying on their small income.

## Conclusions

This paper investigates the impact of the COVID-19 pandemic and related policy measures on the livelihoods of poor and vulnerable households in urban South Africa. We argue that the COVID-19 shock has deepened the economic vulnerability which preceded the crisis. Our qualitative research findings locate this vulnerability at the intersection of three domains. First, the decline in labour earnings and employment prospects; second, the increased exposure to present and future economic shocks; and third, the generalized sense of a loss of individual control and agency brought about by the pandemic, associated with elevated levels of psychological distress.

The intensified sense of powerlessness and heightened vulnerability resulted not only from the sheer magnitude of the economic shock and disruption of the labour market and business activity, but was also determined by the simultaneous undermining of common coping strategies and insurance mechanisms to confront these. The lapse of survivalist livelihood strategies during this crisis, particularly due to the economic disruption of the informal sector, severely deprived the poor and the vulnerable in their ability to secure a living on their own. This was intensified by the co-variate nature of the shock, rendering social networks and informal insurance mechanisms ineffective means of assistance. These combined factors have led to an increased reliance on government grants—the expansions to which during the crisis have been an indispensable element in the livelihood portfolios of the poor.

Our findings give rise to concerns that the COVID-19 pandemic may not only present a temporary income shock but have lasting implications for the incidence, depth, and severity of poverty in South Africa. It may compromise household income-generating activities in the longer term, as the labour market recovery has been incomplete and households have turned to liquidating their small savings and defaulting on insurance payments in the absence of alternative coping strategies. In addition, reduced food consumption in times of hardship, school closures, and the constraints that poor children faced in online teaching may have negative long-term impacts on human capital formation and thus on earnings, thereby deepening existing inequalities and constraining social upward mobility. Through its effects on health, education, and employment prospects, the pandemic may have lasting implications for poverty rates in South Africa.

For the millions of vulnerable South Africans whose livelihoods hang in the balance, an ambitious commitment by the state to confront these challenges is imperative. A few lessons emerge from our research that are instructive in this regard: The usual strategies that households use to cope with shocks are less effective in cases of large co-variate shocks such as those presented by the pandemic. Co-variate shocks require a coordinated response, and only the state has the capacity to provide this relief at the necessary scale. Our research also shows that some of the livelihood effects of the pandemic are likely to endure beyond the acute phase of the public health crisis. This, in addition to South Africa’s existing unemployment crisis, provides a strong case for converting the SRD, which is temporary at present, into a permanent feature of South Africa’s social assistance system.

## Data Availability

The quantitative analysis presented in this paper draws on data from the National Income Dynamics Study: Coronavirus Rapid Mobile Survey (NIDS-CRAM) as well as earlier waves of NIDS collected prior to the pandemic. The data are publicly available here: https://www.datafirst.uct.ac.za/dataportal/index.php/catalog/NIDS-CRAM. https://www.datafirst.uct.ac.za/dataportal/index.php/catalog/NIDS. The qualitative interview data may contain sensitive information about participants and cannot be made publicly available.

## Appendix

**Table A.1:** Respondent characteristics

					2017			2020					
**ID**	**Gender**	**Age**	**Education**	**Birth-place**	**PPI score**	**Estimated likelihood of poverty (UBPL)**	**HH size**	**Access to grant inc. in HH**	**Labour market attachment in Feb**	**HH welfare change Feb-June**	**Associated event Feb-June**	**HH welfare change June-Sep**	**Associated event June-Sep**
R1	Female	62	Secondary incomplete	Cape Town	48	54%	Three	OAP	Pensioneer, living with adult children doing odd jobs	Fall	Loss of labour income within family/household	Fall	Increased distress due to econ. stagnation
R2	Male	59	Primary incomplete	Eastern Cape	36	77%	Three	CSG	Self-employed (street food)	Fall	Reduction in self-employment activities	Fall	Increased distress due to econ. stagnation
R3	Female	54	No Schooling	Eastern Cape	17	99%	Seven or more	CSG	Unemployed, supported by daughter (work at restaurant)	Fall	Loss of labour income within family/household	Fall	Increased distress due to econ. stagnation
R4	Female	61	Primary complete	Eastern Cape	40	74%	Four	CSG/OAP	Pensioneer, with self-employed side activity (street food)	Fall	Break in self-employment activity	Fall	Increased distress due to econ. stagnation
R5	Female	58	Secondary complete (Matric)	Cape Town	57	24%	Five	No	Formal wage employed (primary school teacher)	Fall	No change in formal wage employment, but loss of rental income	Stable	No change
R6	Male	62	Secondary incomplete	Eastern Cape	41	74%	Two	OAP	Doing temporary (piece) jobs	Fall	Loss of temporary (piece) jobs	Stable	Did not resume economic activity
R7	Female	44	Secondary incomplete	Eastern Cape	12	100%	Seven or more	CSG	Informal wage employment (not registered for UIF)	Fall	Laid off from wage job (waiting to resume)	Stable	Did not resume economic activity
R8	Male	36	Secondary incomplete	Cape Town	49	54%	Four	No	Self-employed (irregular)	Fall	Reduction in self-employment activities	Stable	Did not resume economic activity
R9	Male	72	Secondary incomplete	Eastern Cape	24	97%	Six	OAP	Pensioneer household	Stable	No prior reliance on labour market income, only two pensions in the household.	Stable	No change
R10	Male	61	Secondary incomplete	Else-where in South Africa	53	28%	Five	No	Self-employed (home restaurant)	Fall	Reduction in self-employment activities	Rise	Resumption of self-employed activity
R11	Male	42	Secondary incomplete	Eastern Cape	36	77%	Three	CSG	Formal wage employed (grocery store)	Fall	Reduction in hours of formal wage job	Rise	Resumption of full-time wage work
R12	Female	43	Secondary incomplete	Eastern Cape	40	74%	Four	CSG	Part-time wage employed (domestic worker)	Fall	Reduction in hours of wage job	Rise	
R13	Male	30	Secondary complete (Matric)	Cape Town	49	54%	Four	CSG	Formal wage employed (liquor store)	Fall	Reduction in hours of formal wage job	Rise	Resumption of full-time wage work
R14	Male	60	Primary incomplete	Eastern Cape	30	93%	Seven or more	CSG/OAP	Unemployed, supported by wife (domestic worker) and daughter (work at restaurant)	Fall	Loss of labour income within family/household	Rise	Started to receive OAP
R15	Male	52	Secondary incomplete	Eastern Cape	64	11%	Two	CSG	Formal wage employed (painter)	Fall	Laid off from formal wage job (waiting to resume)	Rise	Received UIF/TERS with delay, resumed full-time wage work, and rented out shack in backyard

Notes: The Progress out of Poverty Index (PPI) is calculated based on information collected in the sampling survey. It draws on 15 household-level indicators—including demographics, education, employment, housing, assets, and sanitation—following the scorecard for South Africa. The score is used to evaluate the likelihood of the household falling below the national cost-of-basic-needs upper-bound poverty line (UBPL)

Source: Authors’ compilation

## Footnotes

1 US$ calculated with an exchange rate of US$1 = ZAR17, the approximate value during the first months of the pandemic.

2 At the time of writing, South Africa is in a fifth wave of infections.

3 Established in the early 1980s, Khayelitsha has been growing rapidly, driven by endogenous population growth and continuing rural-to-urban migration. The township comprises old formal areas built originally by the apartheid government, which are generally wealthier, and newer areas that contain a mix of government-provided housing, informal backyard dwellings, and densely populated informal settlements with limited sanitation infrastructure. According to the 2011 Census, 74 per cent of the township population had a monthly household income below ZAR3,200 (equivalent to US$2.1 per person per day in a household of three), thus being considered poor by national and international standards.

4 While no LHI had been conducted with the respondent, extensive background information was available from a structured survey administered in 2017.

5 For simplicity, in the remainder of this paper we refer to the two interview periods as June and September 2020.

6 Interviews took 35 min on average and were conducted in respondents’ native language (isiXhosa) by a skilled and experienced interviewer who had been part of the 2017 research. All interviews were audio recorded and subsequently translated and transcribed into English. The transcripts were entered into NVivo for organization and to facilitate the analysis. Transcripts were read repeatedly by both lead researchers and pertinent sections were clustered together into themes. Within each theme, comparisons were then made across the body of interviews to identify recurring accounts, as well as relevant discrepancies. Each round of interviews was first analysed independently, before comparing and relating themes across the two rounds in a final step of analysis.

7 NIDS is South Africa’s nationally representative panel study, which began in 2008 with a sample of over 28,000 individuals in 7,300 households. It is these individuals that NIDS has followed since 2008. There are currently five waves of data available spanning the nine years from 2008 to 2017, with each wave of data spaced approximately two years apart. NIDS-CRAM draws on the NIDS 2017 sample, but it is a significantly shorter 20-minutes computer-assisted telephone survey. It was designed to rapidly collect data on a broadly representative sample of South African individuals, in order to assess their employment and livelihood outcomes in light of the COVID-19 pandemic.

8 The NIDS-CRAM wave 1 sample was drawn from the adult sub-sample of NIDS 2017 using a stratified sampling design. The final sample size was 17,568 individuals, of which approximately 40 per cent responded, giving a realised sample size of 7,074 completed interviews. The weights in NIDS-CRAM are based on the NIDS 2017 post-stratified weights, adjusted for (i) the probability of being selected into NIDS-CRAM and (ii) non-response (Kerr et al., 2020). The weighted NIDS-CRAM sample can be considered broadly nationally representative of South Africa’s adult population, using NIDS 2017 as a benchmark.

9 Drawing on the definitions used by Statistics South Africa’s (Stats SA), households with a per capita expenditure below the upper bound poverty line, set at ZAR1,136 per person per month in March 2017 Rands (equivalent to US$2.2 per person per day), are classified as poor. Household with a per capita expenditure below the food poverty line, set at ZAR515 (equivalent to US$1 per person per day), are classified as food-poor (Stats, 2017).

10 While some of these losses may be due to factors unrelated to COVID-19, given the timing and the magnitude of the effect, it seems reasonable to assume a direct link in the majority of cases.

11 The GHS is an annual survey of South African households. It collects data on general living circumstances, covering areas such as education, health, housing, access to services, food security, and agriculture.

12 The modelling approach and its applications to NIDS data are described in detail by Schotte et al., (2018) and Zizzamia et. al. (2019), to which we refer interested readers. Underpinning the approach is an understanding of the middle class as an empowered and economically secure part of society that is not only non-poor in the present, but also faces a below average risk of falling into poverty. The approach defines class status at the household level, considering both the current standard of living, measured by household per capita consumption in relation to the poverty line, and the predicted likelihood of sustaining this standard of living in the near-term future, reflected by a poverty risk score. The latter is estimated using a multivariate model of poverty transitions, which considers both the current poverty status and the human, economic and social capital available to the household as determinants of future poverty risks.

13 As before, respondents are classified as poor in 2017 if their household per capita expenditure fell below the national upper bound poverty line. In addition, we define the elite as those with per capita expenditures well above the national average, fixing the elite threshold at two standard deviations above the mean (Schotte et al., 2018).

14 As a preparatory step for this exercise, four FGDs were conducted with participants from the local community. The FGDs were designed to develop a scale capturing social and material welfare levels in the local context, which could be used during the LHIs. This welfare scale was intended to be more subjectively meaningful than narrow, money-metric proxies of income and expenditure, while at the same time facilitating a degree of comparability between cases. To this end, visual aids in the form of four boxes representing different “levels” of society were used during the discussions. Across FGDs, participants emphasized a clear distinction between levels one and two, on the one hand, and levels three and four, on the other, with the latter showing clear markers of poverty: those on level three were characterized as having their most elementary needs (e.g., food and electricity) satisfied, but still needing to economize a lot. All income is used to satisfy other basic needs, without being able to build up a financial cushion to buffer economic shocks. In comparison, those on level four showed markers of food poverty, being repeatedly characterized as “going to sleep on an empty stomach”. While there was some common understanding of the established welfare levels, subjective perceptions regarding own capabilities and position in society also played a role in the individual rankings. For this reason, the approach is well-suited to trace individual shocks and changes to well-being, while comparisons of levels of well-being across individuals must be treated with caution.

15 The Building Industry Bargaining Council negotiates the terms of employment for the industry and administers the industry pension, provident, medical aid, sick and holiday funds.

16 NIDS-CRAM does ask respondents whether “during or since the lockdown started on 27th March [they] received food or shelter” from government, NGOs, churches or other associations, or neighbours or community members. In NIDS-CRAM Wave 1, 9 per cent of respondents report receiving help from neighbours or others in their community. However, since there were no equivalent questions in the pre-COVID NIDS Waves, it is difficult to know whether and which individuals gain or lose access to forms of community-based mutual aid.

17 NIDS-CRAM contained a short module on mental health, including two questions on depressive symptoms taken from the Patient Health Questionnaire (PHQ-2).
